# Molecular mechanisms of docetaxel resistance in prostate cancer

**DOI:** 10.20517/cdr.2020.37

**Published:** 2020-08-21

**Authors:** Yohei Sekino, Jun Teishima

**Affiliations:** Department of Urology, Graduate School of Biomedical and Health Sciences, Hiroshima University, Hiroshima 734-8551, Japan.

**Keywords:** Prostate cancer, docetaxel, drug resistant cancer, biomarker

## Abstract

Docetaxel (DTX) chemotherapy offers excellent initial response and confers significant survival benefit in patients with castration-resistant prostate cancer (CRPC). However, the clinical utility of DTX is compromised when primary and acquired resistance are encountered. Therefore, a more thorough understanding of DTX resistance mechanisms may potentially improve survival in patients with CRPC. This review focuses on DTX and discusses its mechanisms of resistance. We outline the involvement of tubulin alterations, androgen receptor (AR) signaling/AR variants, ERG rearrangements, drug efflux/influx, cancer stem cells, centrosome clustering, and phosphoinositide 3-kinase/AKT signaling in mediating DTX resistance. Furthermore, potential biomarkers for DTX treatment and therapeutic strategies to circumvent DTX resistance are reviewed.

## Introduction

Prostate cancer (PCa) is one of the most common malignancies worldwide. In 2018, it was estimated that there were 1,200,000 new cases and 350,000 men died due to PCa^[1]^. Androgen deprivation therapy (ADT) has been widely utilized as the first-line treatment for PCa^[2]^. Although ADT is initially effective, PCa may eventually progresses to the life-threatening stage of castration-resistant prostate cancer (CRPC)^[3]^. In 2004, docetaxel (DTX) plus prednisone was approved by the FDA as a first-line treatment for patients with CRPC^[4,5]^. DTX is a cytotoxic chemotherapeutic agent which binds to the β-tubulin subunit thereby preventing depolymerization of the microtubules, leading to mitotic arrest, apoptosis, and inhibition of cellular trafficking^[6,7]^. DTX has also been shown to inhibit androgen receptor (AR) nuclear translocation and the expression of AR *in-vitro*^[8,9]^. The recent clinical trials of CHAARTED and STAMPEDE have shown that DTX added to ADT treatment significantly improves the overall survival (OS) of patients with metastatic hormone-sensitive PCa^[10,11]^, indicating that DTX treatment is progressing to becoming crucial in PCa. Nevertheless, despite the prolonged survival resulting from DTX, nearly all patients treated with DTX become refractory due to the development of resistance^[12]^. Therefore, elucidating DTX resistance mechanisms may lead to breakthroughs in CRPC treatment. This review presents molecular mechanisms related to DTX resistance [Table 1], potential biomarkers, and therapeutic strategies to improve survival in CRPC.

**Table 1:** Mechanisms of resistance to DTX in prostate cancer

1. Tubulin alterations^[16-19,21,24]^
The increased expression of βIII-tubulin leads to DTX resistance. βIII-tubulin exhibits predictive value for DTX treatment
2. AR and AR-variants^[25-30,32-39]^
AR signaling and AR-variants are involved in DTX resistance. There are conflicting results regarding AR and AR-variants as biomarkers for DTX resistance
3. ERG rearrangement^[42-47]^
Overexpression of ERG induces DTX resistance by altering microtubule dynamics. Serum TMPRSS2-ERG expression has a role in predictive value for DTX treatment
4. Drug efflux and influx^[48-50,53-57,60-62]^
ABCB1 expression is up-regulated in DTX-resistant PCa cell lines. Several drugs targeting ABCB1 enhance DTX efficacy. SLCO1B3 expression is down-regulated in DTX-resistant PCa cell lines
5. Cancer stem cells^[64,69-74,76-84]^
CD44 and CD133 enrich the stem-cell like properties and contribute to DTX resistance in PCa cell lines
6. Centrosome clustering^[85,88,89,91,92]^
KIF11 and KIFC1 interact with microtubules and are involved in DTX resistance
7. PI3K/AKT signal upregulation^[96-99]^
Long-term DTX therapy induces up-regulated pAKT expression. Several drugs targeting AKT/PI3K enhance DTX efficacy

DTX: docetaxel; AR: androgen receptor; PCa: prostate cancer; pAKT: phosphorylated AKT; PI3K: phosphoinositide 3-kinase

## Mechanisms of resistance to docetaxel in prostate cancer

### Tubulin alterations

The active binding site of DTX to microtubules has been actively studied, in order to elucidate the cause of DTX resistance. Microtubules are formed by the polymerization of a dimer of two globular proteins, α and β tubulin^[13]^. Although seven different β-tubulin isotypes exist in humans, the functional significance of these isotypes has not been fully explained^[14]^. Most mutations in the drug-binding sites are thought to mediate resistance by inducing reduced affinity of the drug-tubulin binding^[15]^. One study has shown that a mutation (F270I) in the drug binding sites of the βI-tubulin gene was discovered in DTX-resistant PCa cell lines, conferring resistance to DTX^[16]^. βIII-tubulin encoded by the TUBB3 gene is a microtubule protein mainly expressed in neuronal cells^[13]^. Some studies have reported that overexpression of TUBB3 confers DTX resistance in PCa^[17,18]^. The status of TUBB3 expression has a predictive value for OS in CRPC patients treated with DTX^[17,19]^. MAPT, which encodes the microtubule-associated protein tau, works in tubulin assembly and microtubule stabilization^[20]^. MAPT expression is upregulated in DTX-resistant PCa cell lines, and subsequent knockdown of MAPT increases the sensitivity of DTX^[21]^. Although these proteins related to microtubules play essential roles in DTX resistance, there has been no effective clinical therapies to target them thus far^[22]^. The formation of microtubule bundling in interphase cells serves as a hallmark of taxane on-target stabilizing activity^[23]^. A recent study showed that loss of microtubule bundling was observed in DTX resistant tumors after DTX treatment using a xenograft model. Notably, despite of the small sample size, the extent of microtubule bundling was associated with DTX response^[24]^.

### AR/AR-variants

Microtubule stabilization plays an important role in AR cellular transport and nuclear translocation^[25]^. DTX activity may partly disrupt AR nuclear transport and signaling^[9,26]^. These findings suggest that AR signaling is involved in DTX resistance. Indeed, several studies have reported that some molecules promote DTX resistance through the activation of AR signaling^[27,28]^. The predictive value of AR using circulating tumor cells (CTCs) has also been reported^[25,29]^. The recent TAXINERGY clinical trial demonstrated that persistent nuclear AR localization in the CTCs under DTX therapy may serve as a predictive marker of DTX resistance^[29]^. In contrast to these reports, sequence analysis has shown that patients with a gain in plasma AR exhibit a favorable response to DTX^[30]^.

AR variants (AR-v7 and AR-v567es) are unable to bind to the ligand dihydrotestosterone. Therefore, AR variants are constitutively active and act to drive PCa progression through promoting transcription of AR target genes^[31]^. Furthermore, AR variants do not require microtubule-assisted translocation due to the lack of microtubule binding domains^[32]^. Thus, it is rational to expect that AR variants are involved in DTX resistance. Several studies have similarly reported that AR variants confer insensitivity to DTX treatment^[32,33]^. However, evidence for the contribution of AR variants to DTX resistance has not been fully established. Some studies have described that AR-v7 does not induce resistance to DTX^[34]^. Moreover, the expression of AR variants is not upregulated in DTX-resistant PCa cell lines^[35,36]^.

Clinical evidence for the contribution of AR variants to DTX resistance also remains conflicting. Recent studies have shown that the increased expression of AR-v7 is associated with a worse prognosis in CTCs expressed in metastatic CRPC treated with taxanes, including DTX^[37,38]^. On the contrary, another study showed no significant association between AR-v7 expression and the efficacy of DTX in CTCs^[39]^. Of note, recent research has shown that AR-v7 exosomal mRNA as quantified by droplet digital polymerase chain reaction is observed in healthy volunteers^[40]^. The non-specific detection of AR-v7 in blood could help to explain the above-mentioned conflicting evidence on the predictive value of AR-v7 to DTX response^[38]^.

### ERG rearrangement

TMPRSS2-ERG rearrangement is a PCa-specific genetic alteration that leads to overexpression of ERG^[41]^. A mass spectrometric analysis has shown that the transcription factor ERG directly interacts with β-tubulin in PCa^[42]^. A recent study on PCa reported an association between ERG fusion status and high expression of TUBB3, which exists as one of the isotypes of β-tubulin^[43]^. Overexpression of ERG induced DTX resistance in CRPC patients by altering microtubule dynamics through interaction with β-tubulin^[44]^. Another recent study found that targeting the TMPRSS2/ERG fusion mRNA using liposomal nano-vectors enhances DTX treatment in PCa^[45]^. Several studies have reported the possible role of TMPRSS2-ERG expression as a biomarker for DTX response. ERG overexpression has a two-fold increase in the chance of developing DTX resistance as compared to ERG-negative cancers in CTCs from CRPC patients treated with DTX^[44]^. Moreover, TMPRSS2-ERG expression in blood and tumor predicts poor OS in metastatic CRPC patients following DTX^[46,47]^. Thus, assessment of TMPRSS2-ERG expression may be a useful tool when selecting treatment in patients with CRPC.

### Docetaxel intracellular accumulation: drug efflux and influx

DTX binds to free tubulin in the cytoplasm, and an adequate intracellular concentration is vital to stabilize the microtubules^[33]^. The intracellular concentration of DTX depends on the ratio of drug influx and efflux pumps^[48]^. Therefore, downregulation of influx transporter activity or upregulation of efflux transporters activity may play crucial roles in DTX efficacy^[49,50]^.

Efflux transporters activity: ATP-binding cassette (ABC) transporters, which transport various molecules across membranes, are classified into the ABC superfamily based on the organization of their ABC domains^[51]^. P-glycoprotein/ATP-binding cassette sub-family B member 1 (ABCB1), which is encoded by multidrug-resistance protein 1, is one of the members of the ABC transporters^[52]^. ABCB1 demonstrates high-affinity binding to DTX and can efficiently pump DTX out of treated tumor cells hence decreasing the efficacy of stabilizing microtubules^[7,53]^. An increase in certain variants of ABCB1 expression has been associated with DTX resistance in PCa^[54]^. Knockdown of ABCB1 increases sensitivity of PCa cell lines to DTX^[49]^. A preclinical study showed that ABCB1 is involved in cross-resistance between DTX and cabazitaxel^[55]^. Several studies have shown the potential of blocking ABCB1 to enhance DTX efficacy through the use of elacridar, BKM1972, enzalutamide, and RORγ antagonist SR2211^[55,56]^. One study investigated the predictive value of ABCB1, and found that exosomal ABCB1 levels are increased in DTX-resistant patients as compared to those in therapy-naïve patients with CRPC^[57]^.

Influx transporter activity: The organic anion transporting polypeptide, which is an SLCO-encoded membrane protein, can transport drugs^[58]^. SLCO1B3 is a known influx transporter of DTX into the cell^[59,60]^. Several studies have shown that SLCO genetic variants (SLCO1B3 and SLCO2B1) are associated with poorer outcomes in PCa^[61,62]^. The expression of SLCO1B3 was significantly downregulated in a DTX-resistant PCa cell line. In addition, overexpression of SLCO1B3 is related to higher intracellular DTX concentrations, suggesting that loss of SLCO1B3 may drive DTX resistance^[60]^.

### Cancer stem cells

Recently, the importance of cancer stem cells (CSCs) has been reported in a wide variety of biological processes relevant to PCa^[63]^. PCa cells surviving chemotherapy exhibit an increased number of CSCs^[63,64]^. CD44, CD133, and ALDH have also been associated as biomarkers for CSCs in PCa^[65-68]^.

CD44: a transmembrane glycoprotein, is a major component of the extracellular matrix and acts as a receptor for several growth factors and cytokines^[69]^. A number of studies have reported that DTX-resistant PCa cell lines are strongly enriched for CD44 expression^[69,70]^. DTX treatment induces a subpopulation of cells which exhibit CD44 upregulation and display enhanced resistance to DTX^[71,72]^. Knockdown of CD44 expression was shown to decrease DTX resistance in PCa cell lines^[69]^. A recent study showed that CD44 promotes migration and invasion of DTX-resistant PCa cells, possibly through the activation of the Hippo-YAP signaling pathway^[69]^, which is a major player in CSCs^[73]^. Another recent study showed that an anti-bacterium, salinomycin, can specifically suppress the tumor-initiating cells, which are enriched for CD44 expression in DTX-resistant PCa cells^[74]^.

CD133: a pentaspan transmembrane protein, has been used as a marker for identification of CSCs in PCa^[75]^. CD133 enriches the stem-cell properties, contributing to DTX resistance in PCa cell lines^[76]^. A recent study showed that the combination of DTX with a nanoplatform targeting CD133 exerts an antitumor effect in a xenograft model^[77]^.

CSC-associated pathways (NOTCH and Hedgehog): Cell lines with DTX resistance display enhanced activity of Notch and Hedgehog signaling^[71,78]^. Knockdown of Notch signaling has been shown to reverse DTX resistance in PCa^[71,79]^. Furthermore, Notch1 signaling promotes DTX resistance via regulating ABCC1 expression in PCa stem cells^[80]^. Inhibition of EGFR and Hedgehog signaling by gefitinib and cyclopamine improves the efficacy of DTX in PCa cell lines^[81,82]^. Recent studies have shown that the Notch pathway inhibitor PF-03084014 and Hedgehog pathway inhibitor GDC-0449 enhance anti-tumoric effects of DTX on PCa^[83,84]^.

### Centrosome clustering

Kinesins are motor proteins that hydrolyze ATP and move along microtubule filaments^[85]^. They are involved in several cellular processes, including that of cellular cargo transport and mitosis^[86]^. In addition, they have been associated with resistance to DTX treatment in solid tumors, including PCa^[85]^. KIF11 separates spindle poles of a mitotic cell by moving to the plus-ends of microtubules^[87]^. KIF11 inhibitors such as ispinesib and S-trityl-L-cysteine have anti-tumor activity in DTX-resistant PCa cell lines^[88,89]^. Kinesin family member C1 (KIFC1) is a minus end-directed motor protein that plays an essential role in centrosome clustering^[85,90]^. Previously, we showed that KIFC1 was associated with a poorer prognosis after radical prostatectomy or after DTX treatment in PCa^[91]^. Furthermore, KIFC1 inhibitor CW069 induces apoptosis and reverses the resistance to DTX in PCa cell lines^[92]^.

### PI3K/AKT signaling

The PI3K/AKT pathway regulates multiple cellular functions through important signaling pathways in cancer^[93]^. The expression of phosphorylated AKT (pAKT) is upregulated in more aggressive subtypes of prostate cancers due to the inactivation of PTEN^[94]^. AKT has also been directly linked to AR signaling, where the blockade of AR leads to activation of AKT^[95]^, indicating that a reciprocal regulation relationship exists between AKT and AR. These findings suggest that the PI3K/AKT pathway may be a promising therapeutic target in CRPC. Furthermore, long-term ADT and DTX induce pAKT expression in patients with CRPC^[96]^, indicating that the AKT pathway is involved in DTX resistance. Indeed, several studies have shown the potential of AKT/PI3K inhibition in CRPC. *In vivo* analysis has shown that the dual PI3K/mTOR inhibitor NVP-BEZ235 sensitizes DTX and has synergistic effect when administered together with DTX in CRPC^[97]^. A further preclinical study has reported that AKT inhibitor AZD5363 has a synergistic effect with DTX in patients with PIK3CA mutation and PTEN mutation^[98,99]^.

### Biomarkers to predict docetaxel anti-tumor activity

As we discover more about DTX resistance, it will become increasingly important to develop novel biomarkers for response to DTX, as well as patient monitoring strategies to stratify patients for treatment.

Circulating tumor cells: CTCs are nucleated tumor cells that are released into the peripheral blood from epithelial tumors^[100]^. The role of CTCs as a prognostic marker in metastatic CRPC patients has been well studied^[101]^. Recent clinical trials have shown that a decrease in CTC count is associated with a favorable outcome in CRPC patients treated with DTX, indicating that the number of CTCs could serve as a prognostic tool for DTX treatment in metastatic CRPC^[102]^. Another recent trial has shown that the CTC count at the start of DTX therapy is an independent prognostic factor in metastatic CRPC treated with DTX. Patients with a low CTC count exhibit a more favorable outcome than those with a high CTC count^[103]^.

Cell free DNA: Apoptotic cells have the potential to release cell-free fragments of DNA (cfDNA) into the bloodstream^[104]^. A retrospective study has shown that the concentration of cfDNA orior to therapy is an independent prognostic marker for response to DTX in CRPC. Patients with low cfDNA concentration demonstrate a more favorable outcome than those with high cfDNA concentration^[105]^. Further studies have supported the concentration of cfDNA as a predictive biomarker of DTX response. The recent FIRSTANA clinical study has shown that a decline in the concentration of cfDNA during the cycles of taxane including DTX correlated with a favorable response^[106]^. Therefore, cfDNA may be of utility in selecting CRPC patients who may benefit from taxane-based chemotherapy.

## Conclusion

Currently, development of resistance to DTX is inevitable in the course of CRPC management. However, several strategies to overcome DTX resistance have been described, and these may improve DTX-based chemotherapy. Further identification of proteins involved in DTX resistance, the establishment of appropriate biomarkers for DTX treatment, and the investigation of therapeutic combination approaches are necessary to prolong patient survival in patients with CRPC.

## Declarations

### Copyright

© The Author(s) 2020.
